# Alterations in Metabolites Associated with Hypoxemia in Neonates and Infants with Congenital Heart Disease

**DOI:** 10.32604/chd.2020.012219

**Published:** 2020-09-07

**Authors:** Evan Pagano, Benjamin Frank, James Jaggers, Mark Twite, Tracy T. Urban, Jelena Klawitter, Jesse Davidson

**Affiliations:** 1 University of Colorado, Department of Pediatrics, Aurora, CO 80045, USA; 2 University of Colorado, Department of Surgery, Aurora, CO 80045, USA; 3 University of Colorado, Department of Anesthesiology, Aurora, CO 80045, USA; 4 Children’s Hospital Colorado Research Institute, Aurora, CO 80045, USA

**Keywords:** Metabolomics, cyanosis, methylmalonic acid, thymine, hypoxanthine, glutamate

## Abstract

**Objectives:**

(1) To measure the global shift in the metabolome in hypoxemic versus non-hypoxemic infants with congenital heart disease; (2) To identify metabolites and metabolic pathways that are altered in hypoxemia.

**Study Design:**

Analysis of serum samples obtained prior to cardiopulmonary bypass from 82 infants ≤120 days old with congenital heart disease requiring surgery at Children’s Hospital Colorado. Infants were divided into groups based on pre-operative oxygen saturations: non-hypoxemic (>92%), mild hypoxemia (85–92%), and severe hypoxemia (<85%). Tandem mass spectrometry was used to analyze 165 targeted metabolites. Partial least squares discriminant analysis and *t*-tests were used to determine differences among metabolic profiles and individual metabolites respectively.

**Results:**

The broad metabolic fingerprint of neonates or older infants did not vary by degree of hypoxemia. There were 12 individual metabolites that differed between hypoxemic and non-hypoxemic neonates, including lower methylmalonic acid (*p* = 2.44 × 10^−4^), glutamate (*p* = 0.001), and hypoxanthine (*p* = 0.003), and higher thymine (*p* = 8.67 × 10^−4^) and myo-inositol (*p* = 0.014) seen in hypoxemic neonates. Individual metabolites did not vary significantly between older infants with or without hypoxemia.

**Conclusions:**

We did not find evidence supporting global metabolic changes associated with cyanotic congenital heart disease in neonates or older infants. However, specific metabolites did discriminate between hypoxemic and non-hypoxemic neonates. These include methylmalonic acid, as well as several metabolites known to change in hypoxia-reoxygenation states (hypoxanthine) and chronic hypoxemic states (glutamate, thymine, myo-inositol) and may represent specific metabolic changes triggered by hypoxemia among neonates with cyanotic congenital heart disease.

## 1 Introduction

Metabolites are low molecular weight compounds that are key drivers of cellular physiology [1–3]. The collection of metabolites, also known as the metabolome, is constantly changing and determines the minute-to-minute phenotype of the cell [4]. Evaluating the global metabolome can provide insight into changes in response to external conditions and can create a picture of the metabolic environment in cells, tissues, or other fluids [1–3,5].

Cardiovascular disease is known to affect both the global metabolic profile and individual metabolites in adults [3,6–8], however less is known about the effects of cardiovascular disease on the metabolome in children. We and others have previously demonstrated significant changes in the metabolome following pediatric cardiac surgery with cardiopulmonary bypass (CPB) for repair of congenital heart disease (CHD); these changes are likely related to the substantial physiologic disruption from surgical trauma, hemolysis, systemic inflammatory response, and ischemia-reperfusion injury [2,9–11]. Prior studies have looked at the metabolome in subjects with acute tissue hypoxia [12] and demonstrated changes in the arginine/nitric oxide (NO) pathway in acute hypoxemia due to respiratory causes [13], but no study has evaluated the effects of chronic hypoxemia on the larger metabolome. Since infants with cyanotic CHD live with varying degrees of hypoxemia based on the relative proportion of systemic and pulmonary blood flow, we sought to assess how hypoxemia may affect the metabolome in these patients, and whether those changes differed in neonates compared to older infants.

Our hypothesis-generating study sought to explore metabolite alterations that may occur in the setting of CHD with hypoxemia (cyanotic heart disease). Our study used targeted metabolic fingerprinting and individual metabolite analysis to identify differences in the preoperative circulating metabolome of neonates and young infants with CHD living with various degrees of hypoxemia. The identification of specific metabolites that are altered in patients with cyanotic CHD is the first step to determine the effects of hypoxemia on the metabolic profile. These individual metabolites would serve as potential targets for future quantitative, integrative, and mechanistic studies in patients with this cyanotic phenotype [14].

## 2 Methods

### 2.1 Clinical Cohort

This study was a secondary analysis of residual frozen serum samples from a previously published prospective cohort study, which looked at changes in alkaline phosphatase activity in infants undergoing cardiothoracic surgery with CPB. Methods for the parent cohort study and metabolomics analyses were previously published [4,10]. The study enrolled infants ≤120 days of age scheduled to undergo cardiothoracic surgery with CPB. Residual samples used for the current analysis were drawn preoperatively (after induction of anesthesia and before the first surgical incision). This study was performed as part of a pre-specified secondary analysis of circulating metabolites in this cohort. Subjects were included in this secondary analysis if residual frozen serum samples were available from the pre-operative time point (immediately after induction of anesthesia and central line placement). The Colorado Multiple Institution Review Board approved the protocol and informed written consent was obtained from the subjects’ family prior to enrollment.

### 2.2 Clinical Data

Baseline perioperative clinical data were collected on all subjects, including sex, age at surgery, and pre-operative oxygen saturation. Age at surgery was used to define neonates (≤30 days of age) and infants (31–120 days of age). Our previous analyses have shown that there are age-related changes in the metabolomic fingerprint [4]; therefore, we examined neonates and infants separately. Pre-operative oxygen saturations (SpO_2_) were collected using the last documented SpO_2_ prior to the subject entering the operating room. Saturations were divided into three groups based on accepted in clinical practice at our institution. Non-hypoxemia was defined as SpO_2_ >92%, mild hypoxemia as SpO_2_ 85–92%, and severe hypoxemia as SpO_2_ <85%. The primary outcome was the global difference in the circulating metabolic profile between hypoxemic and non-hypoxemic subjects. The secondary outcome was the difference in expression of individual metabolites between hypoxemic and non-hypoxemic subjects.

### 2.3 Sample Collection and Processing

Serum samples were obtained prior to subjects undergoing CPB. All samples were processed in a standardized manner per the study protocol. Briefly samples were collected in red-top tubes and allowed to clot for 30 minutes centrifuged at 3000 rpm (1734 g) for 10 minutes at room temperature. Then, serum aliquots were placed in standard cryovials immediately frozen at −70°C for storage and subsequent batch analysis [4]. Samples underwent no more than a single freeze-thaw cycle. These processing and storage methods are consistent with current published recommendations for optimizing serum metabolomics analyses [15,16].

### 2.4 Metabolomics Assay

As was previously described for the parent investigation, sample analysis was performed on the basis of a previously validated approach using targeted liquid chromatography—tandem mass spectrometry [17,18]. Briefly, 50 μL serum sample was combined with 400 μL cooled methanol, incubated for protein precipitation, dried in a centrifuge, reconstituted using 20 μL water/methanol (80:20), and subjected to a modified targeted metabolomics analysis with relative quantification [4]. The current assay measured 165 metabolites. Precursor ion and fragment ion transitions, metabolite names, dwell times, and appropriate collision energies for both positive and negative ion modes were adapted from Yuan et al. [17]. Data acquisition was performed using an Agilent 1200 series high-performance liquid chromatography (HPLC) system (Agilent Technologies, Palo Alto, CA) interfaced with an ABSciex 5500 hybrid triple quadrupole/linear ion trap mass spectrometer (Concord, ON, Canada) [4]. MultiQuant, v2.1.1. (ABSciex) software was used for initial data processing and the resulting metabolite peak areas were normalized to the area of the internal standard and tissue weight; this ratio was used for all subsequent statistical analysis [4].

### 2.5 Statistical Analysis

Metabolomic statistical analysis was performed using Metaboanalyst 4.0, a web-based metabolomics analysis tool [19]. Individual .csv files were submitted to the program and data underwent log transformation and auto-scaling (mean-centered and divided by the standard deviation of each variable). Partial least squares discriminant analysis (PLS-DA) was a supervised method of multivariable analysis used to determine if there was any detectable difference in the global metabolic profile between pre-defined groups. R^2^ values describe the predictive accuracy of the model and range from 0 to 1; the closer to 1, the higher the predictive accuracy. Q^2^ values indicate goodness of fit; negative Q^2^ values indicate that the data is overfitted (i.e., even with no actual difference, the program will still discriminate between groups by picking some features that are ‘different’ by pure chance) [19]. PLS-DA was used to look for global differences between groups and Variable Importance in Projection (VIP) scores depicted which individual metabolites contributed most to the variation between groups; VIP scores greater than or equal to 2.0 were considered important. Biomarker analysis with student *t*-tests compared specific metabolites’ relative concentrations between hypoxemic and non-hypoxemic groups. Since this was a hypothesis-generating profiling study aimed at identifying candidate metabolites for future detailed analyses, we reported any metabolite whose relative level between groups was statistically significant at an unadjusted *p*-value of <0.05 and a false discovery rate (FDR) of <0.25, such that 3 out of every 4 metabolites identified was a true positive finding while expecting 1 out of every 4 metabolites identified to be a false discovery [14].

## 3 Results

### 3.1 Subjects and Clinical Characteristics

A total of 122 patients were enrolled in the parent cohort study. Eighty-two subjects had a preoperative serum aliquot available for metabolomic analysis; of these, 40 were infants and 42 were neonates. One neonatal subject was excluded from the analysis as a severe outlier with 10-fold differences in multiple metabolites likely due to matrix interference. Baseline characteristics are shown in (Tab. 1). As expected, the infant group demonstrated significantly higher weight and a significantly lower hematocrit (secondary to the physiologic nadir). The infant group also had a higher proportion of premature births, likely due to our clinical practice of delaying surgery for premature infants until after the neonatal period when possible. Of the 40 infants, 22 were non-hypoxemic, 9 were mildly hypoxemic, and 9 were severely hypoxemic. Of the 41 neonates that were included in the final analysis, 18 were non-hypoxemic, 14 were mildly hypoxemic, and 9 were severely hypoxemic.

### 3.2 Preoperative Metabolic Fingerprint of Older Infants

**Figure 1:**
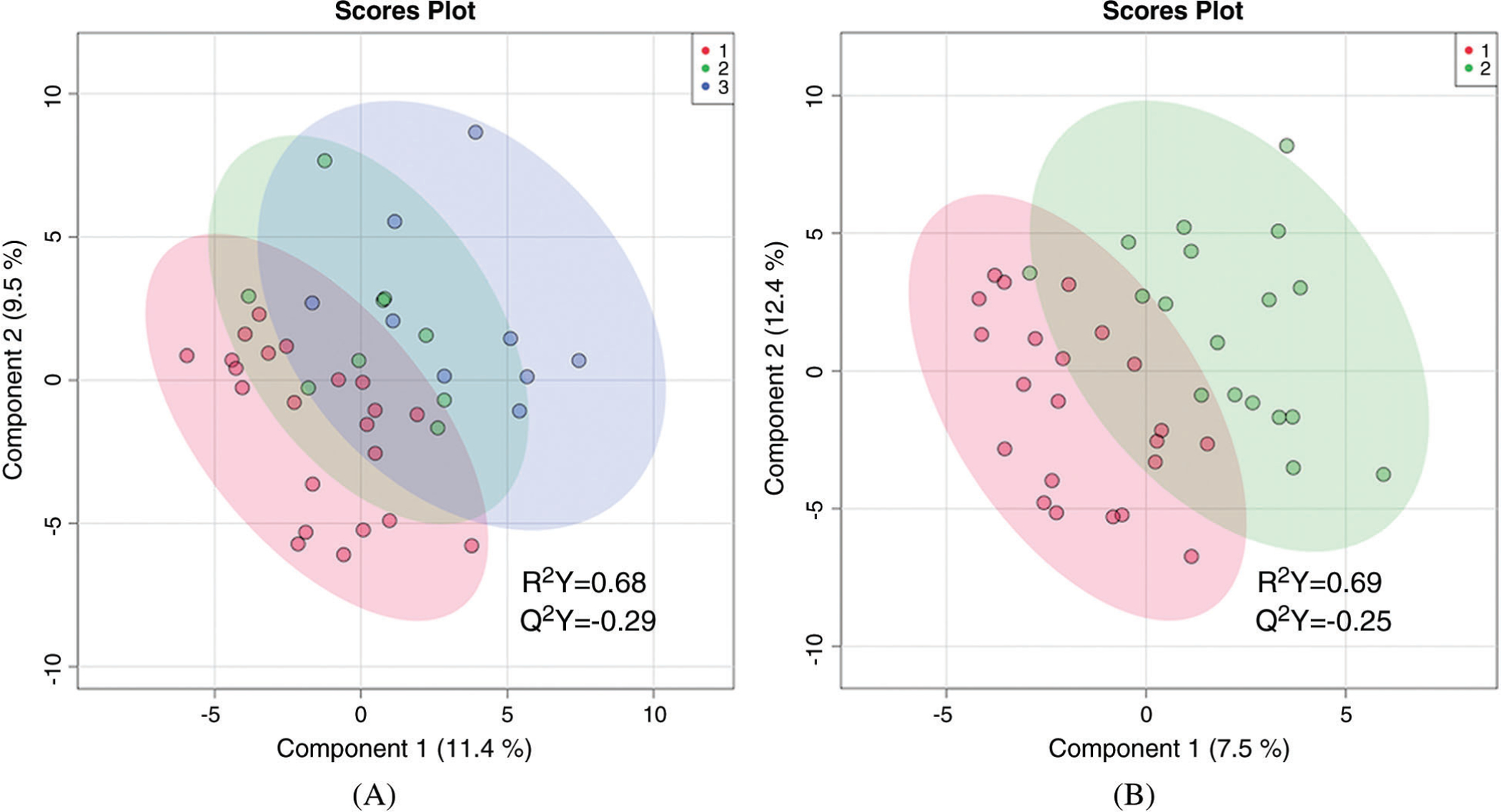
Two-dimensional partial least squares—discriminant analysis comparing metabolic fingerprints of older infants using three categories of hypoxemia (A) where red = no hypoxemia, green = mild hypoxemia, blue = severe hypoxemia; and two categories of hypoxemia (B) where red = no hypoxemia and green = any hypoxemia

There was no statistically significant difference in the global metabolic profile of non-hypoxemic, mildly hypoxemic, and severely hypoxemic infants. PLS-DA discriminated between these three groups, but the data were overfitted (R^2^ = 0.68, Q^2^ = −0.29) as shown in (Fig. 1A). When mildly and severely hypoxemic infants were combined into one group (termed hypoxemic infants), there was no statistically significant difference in the global metabolic profile of hypoxemic and non-hypoxemic infants (R^2^ = 0.69, Q^2^ = −0.25) as shown in Fig. 1B. There were no individual metabolites that differed in a statistically significant way between hypoxemic and non-hypoxemic infants, with an FDR of <0.25.

### 3.3 Preoperative Metabolic Fingerprint of Neonates

**Figure 2:**
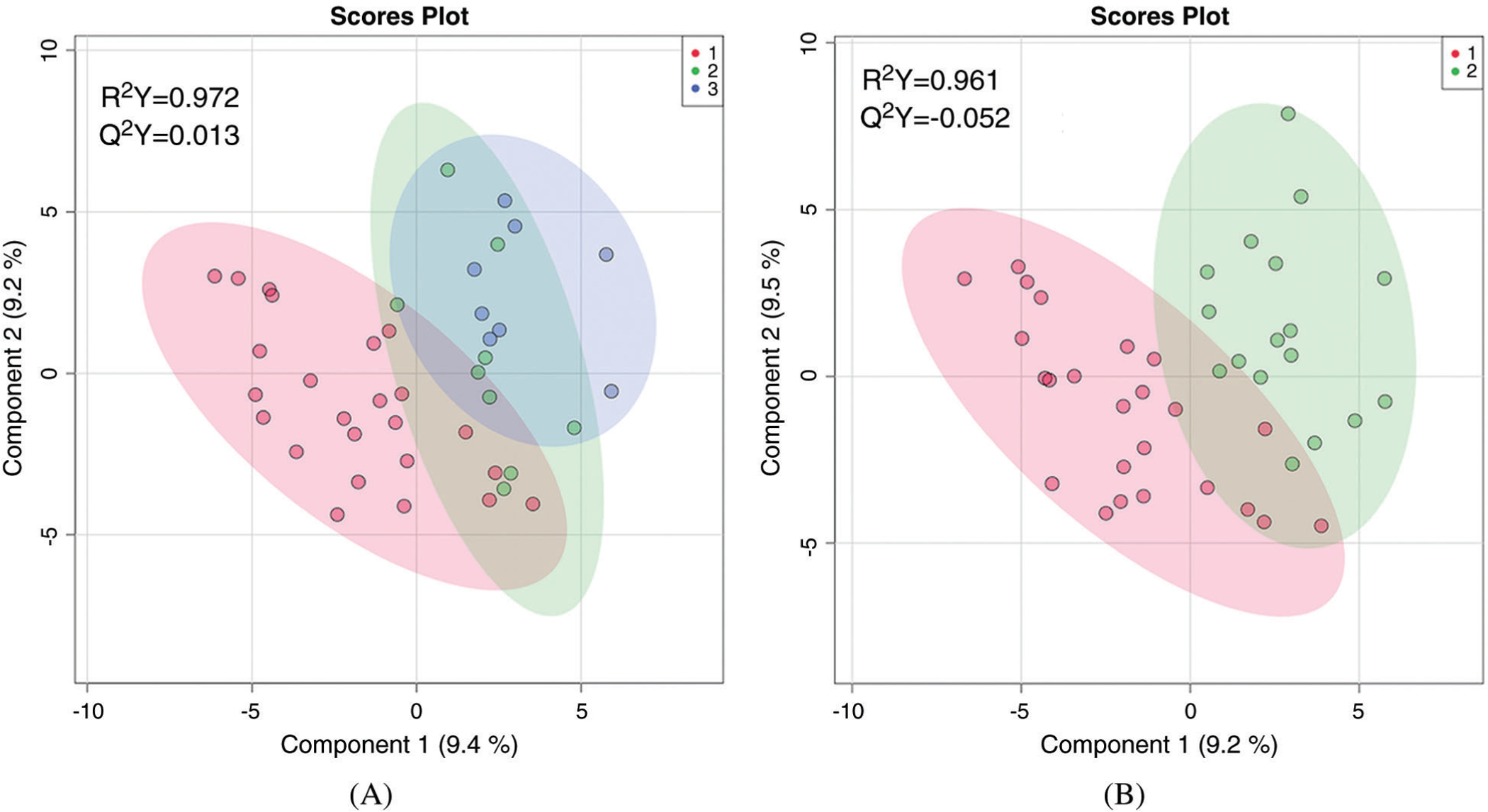
Two-dimensional partial least squares—discriminant analysis comparing metabolic fingerprints of neonates using three categories of hypoxemia (A) where red = no hypoxemia, green = mild hypoxemia, blue = severe hypoxemia; and two categories of hypoxemia (B) where red = no hypoxemia and green = any hypoxemia

First, the global metabolic profiles of non-hypoxemic, mildly hypoxemic, and severely hypoxemic were compared. PLS-DA was able to distinguish between these three groups (R^2^ = 0.972) but the data were overfitted (Q^2^ = 0.013), as shown in Fig. 2A. With the mildly and severely hypoxemic neonates combined (termed hypoxemic neonates), there remained difference in the global metabolic profile of hypoxemic versus non-hypoxemic neonates (R^2^ = 0.961), but once again the data was overfitted (Q^2^ = −0.052) as shown in (Fig. 2B).

### 3.4 Metabolite Differences in the Neonatal Cohort

**Figure 3:**
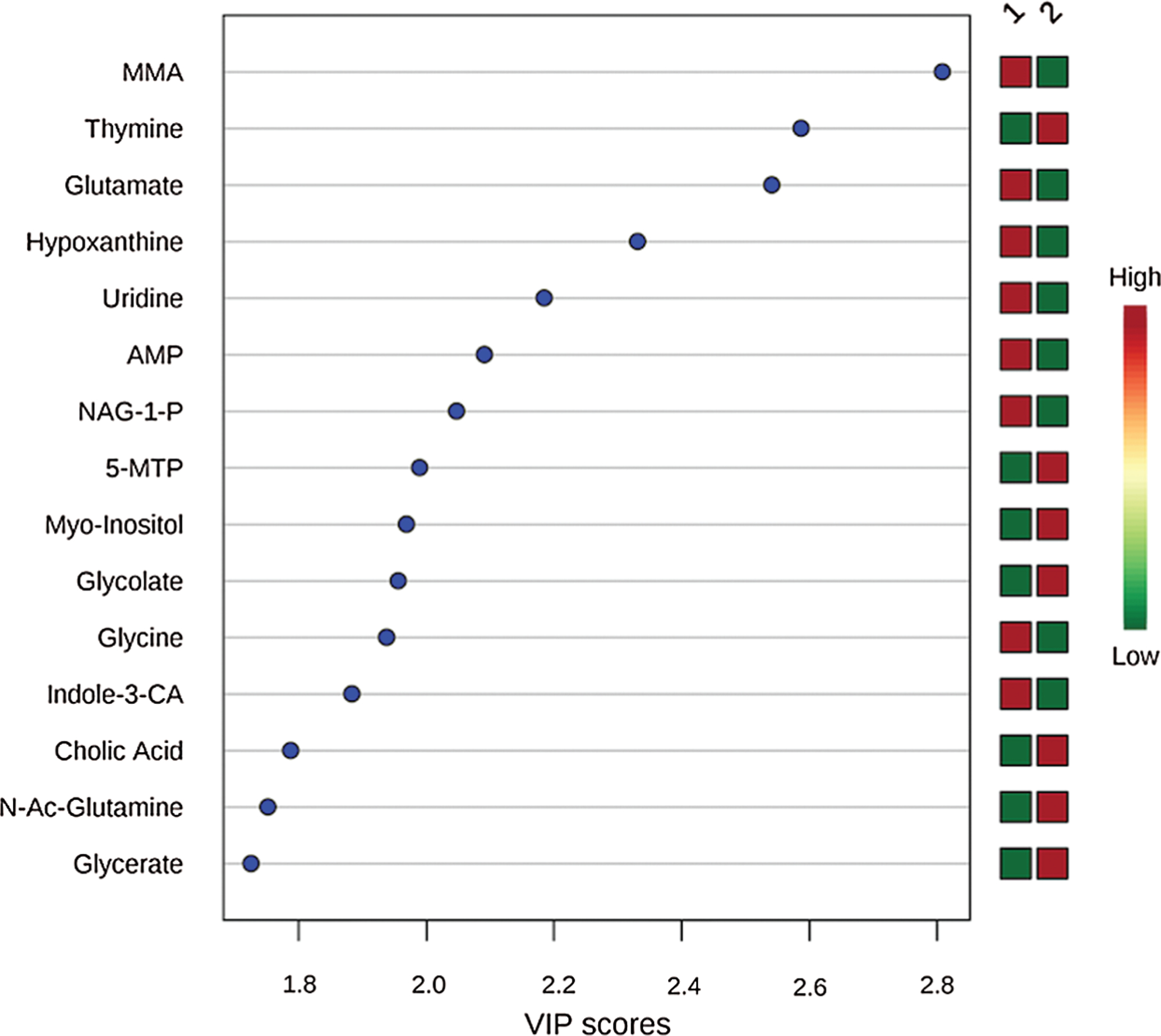
Variable Importance in Projection (VIP) scores for the top 15 metabolites contributing to variation in preoperative metabolic fingerprints of non-hypoxemic and hypoxemic neonates. MMA indicates methylmalonic acid; AMP, adenosine monophosphate; NAG-1-P, N-acetyl-glutamine-1-phosphate; 5-MTP, 5-methoxytryptophan; indole-3-CA, indole-3-carboxylic acid; N-Ac-Glutamine, N-acetyl-glutamine

**Figure 4:**
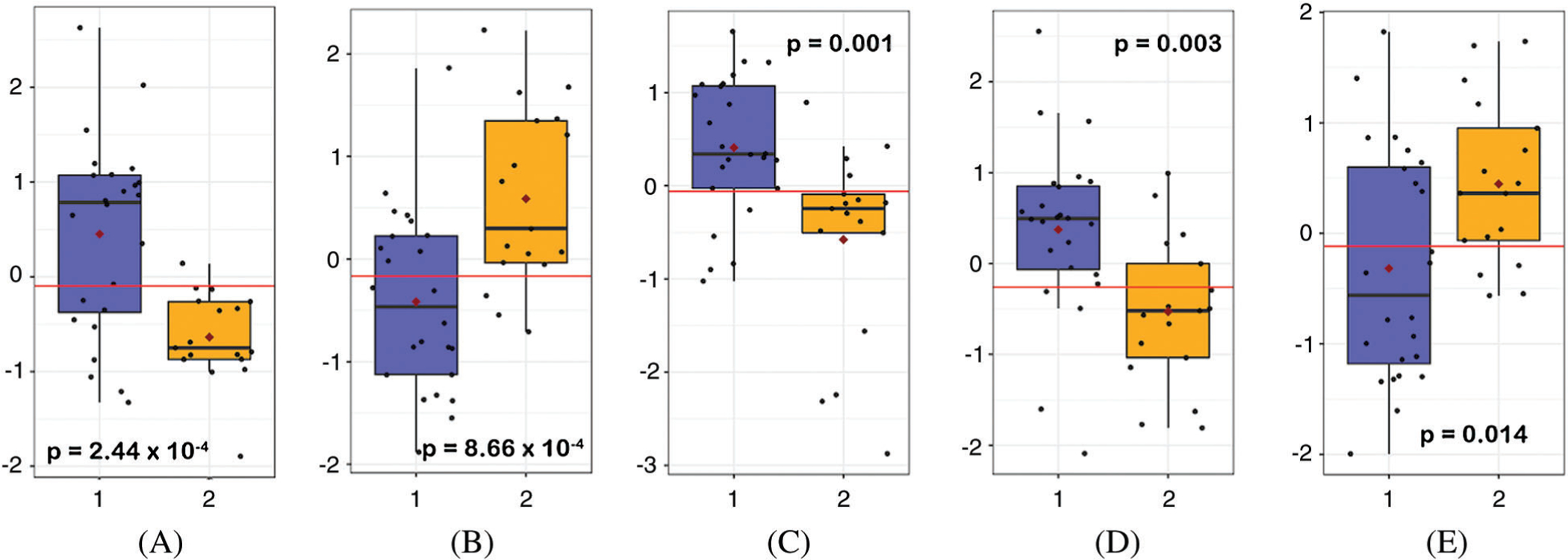
Normalized preoperative concentrations of methylmalonic acid (A), thymine (B), glutamate (C), hypoxanthine (D), and myo-inositol (E) in non-hypoxemic (blue) and hypoxemic (orange) neonates

The top 15 metabolites that contributed most to the differences between hypoxemic and non-hypoxemic subjects are shown in Fig. 3. Of those, there were 12 metabolites that were identified to be significant with an unadjusted *p*-value of <0.05 and an FDR of <0.25. Of those 12, there were 8 metabolites whose levels were lower in hypoxemic neonates, including methylmalonic acid (MMA, *p* = 2.44 × 10^−4^), glutamate (*p* = 0.001), hypoxanthine (*p* = 0.003), uridine (*p* = 0.006), adenosine monophosphate (AMP, *p* = 0.009), N-acetylglucosamine-1-phosphate (NAG-1-P, *p* = 0.01), glycine (*p* = 0.016), and indole-3-carboxylic acid (indole-3-CA, *p* = 0.019). There were 4 metabolites whose levels were higher in hypoxemic neonates, including thymine (*p* = 8.66 × 10^−4^), 5-methyoxytryptophan (5-MTP, *p* = 0.013), myo-inositol (*p* = 0.014), and glycolate (*p* = 0.015). Relative normalized levels of five metabolites are shown in (Fig. 4). These findings remained unchanged after also controlling for single ventricle physiology, pulmonary over-circulation, and gender.

**Figure 5:**
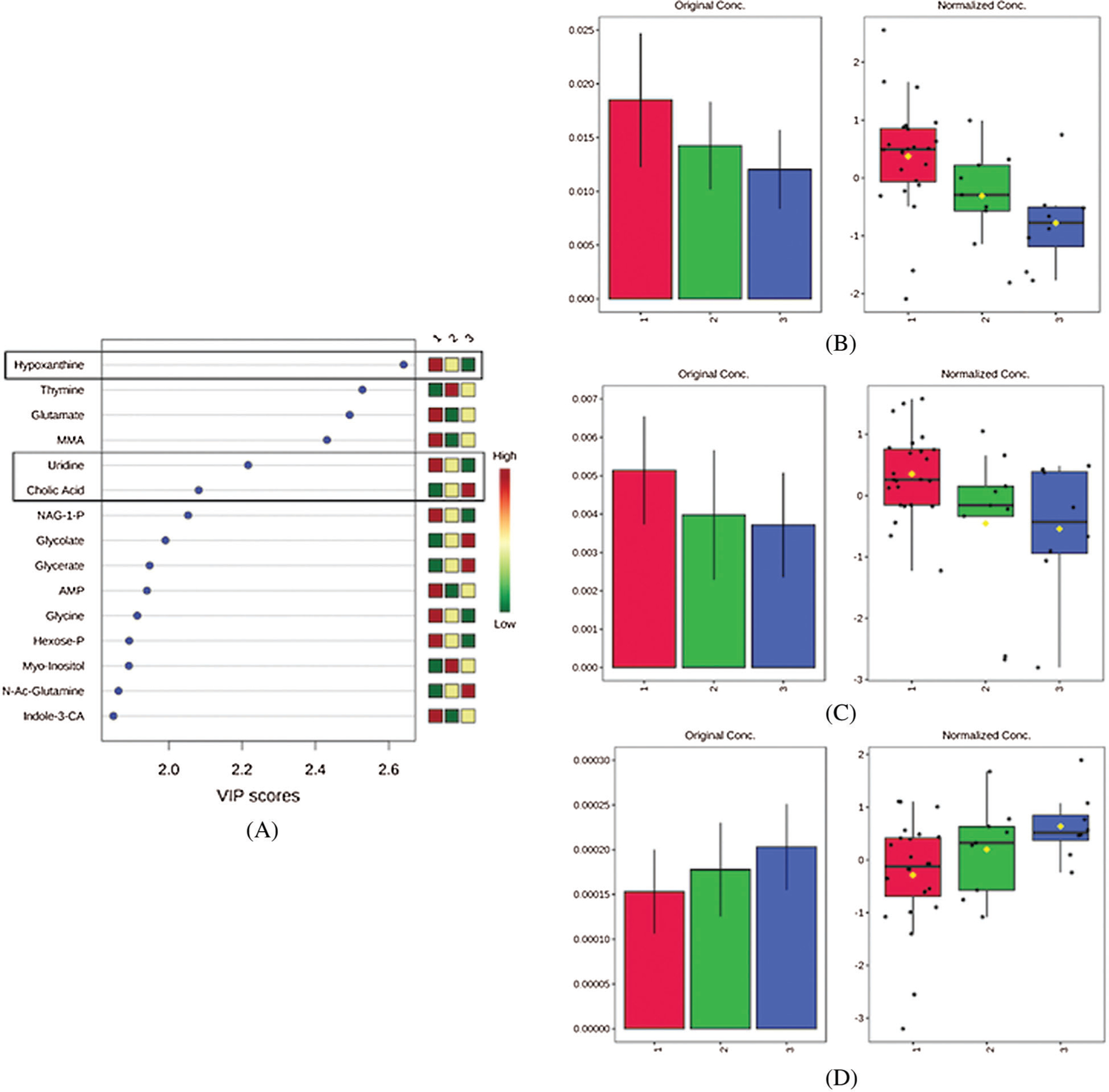
Variable Importance in Projection (VIP) scores for the top 15 metabolites contributing to variation in preoperative metabolic fingerprints of non-hypoxemic, mildly hypoxemic, and severely hypoxemic neonates (A). Normalized concentrations of hypoxanthine (B), uridine (C), and cholic acid (D) that vary with degree of hypoxemia where red = no hypoxemia, green = mild hypoxemia, and blue = severe hypoxemia. MMA indicates methylmalonic acid; NAG-1-P, N-acetyl-glutamine-1-phosphate; AMP, adenosine monophosphate; Hexose-P, hexose-phosphate; N-Ac-Glutamine, N-acetyl-glutamine; indole-3-CA, indole-3-carboxylic acid

Although the global metabolic profile of non-hypoxemic, mildly hypoxemic, and severely hypoxemic neonates did not differ in a statistically significant way, there were several individual metabolites that appeared to have a dose-response relationship with degree of hypoxemia. These included hypoxanthine, uridine, and cholic acid. Relative levels of hypoxanthine and uridine decreased as degree of hypoxemia worsened, while relative levels of cholic acid increased as degree of hypoxemia worsened (Fig. 5).

## 4 Discussion

### 4.1 Key Findings

Our investigation was a hypothesis-generating study and was therefore designed to be as inclusive as possible, using targeted metabolomics to identify metabolites that could be of biologic or clinical significance in the aberrant phenotype, hypoxemia [14]. Many patients with CHD live with chronic hypoxemia, but the potential metabolic consequences of this hypoxemic state remain unclear. If there are differences in metabolites in hypoxemic versus non-hypoxemic patients with CHD, could these differences also have untoward biologic effects that could help explain some of the long-term sequelae of cyanotic CHD? This metabolic profiling study represents the first step in identifying differentially produced metabolites that could be future targets for mechanistic and potentially therapeutic studies to modulate the effects of chronic hypoxemia.

In this study, we found that we could not reliably distinguish between hypoxemic and non-hypoxemic subjects (neonates or older infants) based solely on their global metabolic profile. Individual metabolites, however, proved more informative particularly in the neonatal portion of the cohort. Among neonates, there were 12 metabolites whose normalized relative levels differed significantly between normoxemic and hypoxemic patients with a *p*-value <0.05 and an FDR of <0.25. Of those 12, MMA, thymine, and glutamate were the most significantly altered metabolites between hypoxemic and non-hypoxemic neonates. Notably, the differences observed in the neonatal cohort were not observed in older infants. Further research is needed to understand the significance of this difference by age, but our data suggest that the older patients could have sufficient time to achieve metabolic homeostasis in the face of chronic hypoxemia, potentially through unexplored changes at the gene/protein expression level.

Because the use of metabolomic techniques in patients with CHD is a recent development, there are few studies available for direct comparison. To our knowledge there are no studies that have taken a metabolomic approach to the evaluation of the effects of hypoxemia on CHD patients. In order to explore the potential biologic relevance of our findings, we had to leverage disease states that could be comparable to the hypoxemia observed in patients with CHD. We acknowledge that there are limitations to this approach, but because the goal of our study was to look for trends in metabolites that could serve as the basis for future studies, we felt it important to explore the literature broadly and compare our findings, even if the pathophysiology studied differed significantly.

### 4.2 Methylmalonic Acid

We report that levels of MMAwere lower in hypoxemic neonates compared to non-hypoxemic neonates. Very little is known about the relationship of MMA to hypoxemia, but one recent mouse study demonstrated changes in metabolite concentrations (cobalt and MMA) in the white matter of mice exposed to long-term intermittent hypoxia (LTIH) [20]. LTIH damages white matter by disrupting myelination. They found higher concentrations of cobalt (a surrogate measure for vitamin B_12_) within injured white matter and hypothesized that the brain may sequester vitamin B_12_ to help repair tissue damage associated with oxidative injury in LTIH. They looked at both plasma MMA levels as well as white matter cross-sections and found that plasma MMA levels were lower across all LTIH mice, compared to mice not exposed to LTIH [20]. Since higher levels of MMA can indicate a relative vitamin B_12_ deficiency, the lower levels of MMA observed in damaged white matter were thought to suggest a relative excess of vitamin B_12_, which fits with their hypothesis that the brain is sequestering vitamin B_12_ to help with tissue repair [20]. Our data also demonstrated lower serum levels of MMA in hypoxemic neonates compared to non-hypoxemic neonates, and we hypothesize that a similar mechanism involving vitamin B_12_ sequestration may be responsible for the lower serum levels of MMA observed in our hypoxemic neonates. Future studies looking at both MMA and vitamin B_12_ levels in hypoxemic neonates alone could be useful in determining what enzymes within this metabolic pathway may be altered as a result of hypoxemia.

### 4.3 Thymine and Pyrimidine Metabolism

We found that our hypoxemic neonates had higher normalized levels of thymine compared to non-hypoxemic neonates. This finding is consistent with previous studies exploring thymidine phosphorylase (TP) activity in hypoxic environments. TP, also known as platelet-derived endothelial cell growth factor (PD-ECGF), is highly expressed in solid tumors. It has been shown to have both anti-apoptotic and pro-angiogenic effects [21–23]. In addition, TP phosphorylates thymidine to create thymine, which is important in pyrimidine salvage and ensures a sufficient pool for DNA repair and replication [22]. Kitazono et al. examined the effects of hypoxia on solid tumor cells that expressed PD-ECGF/TP to see whether expression of these factors affected tumor survival. They found that hypoxic conditions induced PD-ECGF/TP, leading to increased TP activity and higher levels of thymine. As a solid tumor grows, its center has decreased blood supply and is therefore exposed to hypoxia, which leads to central necrosis. They proposed that increased activity of TP represents an adaptive mechanism (that has yet to be defined) of tumor cells to resist apoptosis in order to maintain tumor progression [21]. It is possible that our hypoxemic infants also have increased thymine secondary to changes in TP activity, with potential implications on angiogenesis, apoptosis, and DNA replication/repair [22]. These findings indicate the need to assess TP activity and gene expression in the CHD population as areas of future research, as well as the cell types responsible for thymine production.

### 4.4 Glutamate

We found that hypoxemic neonates had relatively lower levels of glutamate compared to non-hypoxemic neonates. Previous studies have shown that there is a relationship between hypoxia and glutamate [24–26]. Glutamate is a non-essential amino acid and is an excitatory neurotransmitter in the central nervous system (CNS) of mammals [26]. Within the CNS, astrocytes are neuronal cells whose fundamental role is uptake of glutamate to aid in the prevention of excitotoxicity that is caused as a result of excessive glutamate [24,26]. Glutamate homeostasis helps to prevent neuronal death after hypoxic/ischemic episodes [24]. Review of the literature suggests that different hypoxemic environments seem to result in different glutamate responses [24,25,27]. Previous studies have found that hypoxia can induce the release of glutamate [25], which leads to neuronal damage. Dallas et al. examined how hypoxia affected the uptake of glutamate in order to better understand the relationship between hypoxia and glutamate-induced excitotoxicity. They assessed glutamate transporter activity and transporter expression in astrocytes exposed to hypoxic conditions, and found that hypoxia led to decreased glutamate transporter protein expression, and did not affect the activity of transporters already expressed. Fewer transporter proteins lead to higher extracellular levels of glutamate [24]. Animal studies examining the relationship between hypoxia and extracellular glutamate in rat pheochromocytoma cells demonstrated decreased extracellular glutamate [27,28]. One study examined the effects of hypoxia on glutamate metabolism and uptake in rat pheochromocytoma cells; researchers found that hypoxia induced changes in cellular enzymes involved in both glutamate transport and metabolism, and the combination of these changes led to an overall decreased concentration of extracellular glutamate [27]. In addition, previous studies have shown that erythropoietin (Epo) has a neuro-protective effect in both hypoxia-induced and glutamate-induced neuronal damage [29]. A study by Lourhmati et al. examined the effects of Epo on reducing the effects of excessive extracellular glutamate in aged rat glial cells. They found that Epo increased glutamate uptake by astrocytes, thus decreasing the concentration of extracellular glutamate in these rat brains [28]. This finding is particularly interesting and potentially applicable to our patients, who likely have increased circulating Epo as a result of chronic hypoxemia. Several of these studies primarily focused on glutamate uptake in neuronal cells, and it is unknown whether hypoxia has a similar effect on glutamate uptake or release in other cells of the body. Also, these studies looked at tissue hypoxia, whereas we were exploring neonates with hypoxemia (who typically did not have true tissue hypoxia). Finally, glutamate is involved in a variety of metabolic pathways, both as a substrate and a product [26] and these studies did not look at the broad effect of hypoxia on those other pathways. More detailed analysis of glutamate metabolism in other types of cells (e. g., cardiac cells obtained in tissue biopsy), different hypoxia models, and with an emphasis on downstream signaling may be useful in helping to better understand the clinical importance of the lower levels of glutamate that we observed in our hypoxemic neonates. Since previous studies have found a relationship between Epo and glutamate, future studies directly correlating circulating Epo and glutamate in our patients are also warranted, as are translational models evaluating Epo modulation in chronic hypoxemia without hypoxia.

### 4.5 Hypoxanthine and Purine Metabolism

Among our neonates, we found that patients with normoxemia had higher normalized concentrations of hypoxanthine compared to those with hypoxemia. The relationship between hypoxanthine and hypoxia has been extensively studied, and hypoxanthine has found to be associated with acute ischemia-reperfusion injury [30,31]. Hypoxanthine is involved in the purine salvage pathway, which permits the regeneration of purines without having to go through de novo synthesis. In this pathway, hypoxanthine is converted to inosine monophosphate (IMP) and can subsequently be converted to guanine monophosphate (GMP) or adenine monophosphate (AMP) [30]. If hypoxanthine is not used for purine salvage, it is degraded by xanthine oxidase (XO), which leads to the production of uric acid and reactive oxygen species (ROS) [31]. Under hypoxic conditions, purine salvage is reduced, which leads to the accumulation of hypoxanthine. When hypoxanthine accumulates in tissues and then we treat the patient’s hypoxia with oxygen, this leads to the generation of ROS and tissue damage [31–33].

Contrary to the findings in acute hypoxia/reoxygenation, our research found that neonates with hypoxemia actually had lower normalized concentrations of hypoxanthine. It could be hypothesized that many of our hypoxemic neonates were placed on supplemental oxygen, driving the degradation of hypoxanthine and the production of ROS. Alternatively, another study found that hypoxanthine levels were lower in both human and mouse red blood cells (RBCs) exposed to chronic hypoxia [30]. They proposed that chronic hypoxia *in vitro* prevents the accumulation of hypoxanthine by decreasing AMP deamination, rather than by promoting salvage reactions [30]. AMP deaminase has also been shown to decrease in cardiac ischemia and AMP deaminase inhibition may be protective [34,35]. Thus it is possible that neonates with cyanotic CHD have developed similar compensatory changes reducing AMP deamination to prevent excessive hypoxanthine accumulation and adenine nucleotide depletion.

### 4.6 Inositol Phosphate Metabolism

Our hypoxemic neonates had higher relative levels of myo-inositol compared to non-hypoxemic neonates. Inositol is the structural basis for numerous derivatives that regulate a multitude of cellular processes, including gene transcription, vesicular trafficking, intracellular calcium signaling, and the growth and development of neurons [36]. In the nervous system, many different types of cells respond to external stimuli via inositol lipid signaling pathways [37]. Inositol and its derivatives also play a role in moderating glucose metabolism, and alterations in inositol metabolism have been associated with insulin resistance [38]. Myo-inositol is also thought to play a role in respiratory neural control, and decreased levels have been associated with neonatal apnea [36]. Another study found that metabolites of inositol are neural stimulants [37]. Although the exact mechanisms are unknown, the higher relative levels of myo-inositol in our hypoxemic neonates could indicate alterations in metabolic pathways that would confer a protective effect. These could include alterations in glucose metabolism to help with optimizing energy utilization, or alterations in pathways that could clear ROS in these hypoxemic neonates. In addition, higher levels of myo-inositol could also lead to faster respiratory rates as a response to hypoxemia.

### 4.7 Limitations

There are several limitations in our study. First, only 165 metabolites were considered in the current assay, so it is unknown whether other metabolites/metabolic pathways could provide better discrimination between infants based on degree of cyanosis or increased mechanistic understanding of the metabolic response to hypoxemia. Future quantitative and pathway mapping studies should be performed targeting the pathways identified in this pilot study for comprehensive analysis. Second, we used cutoffs to determine hypoxemia and non-hypoxemia based on clinical practice at our institution. However, it is unclear if metabolic changes follow our clinical practice or if there is a continuous versus threshold effect. Larger studies are needed to determine which metabolites demonstrate a dose-response effect with hypoxemia. Third, our study did not account for the level of oxygen support that each patient received. We chose to examine the oxygen level achieved by the clinical care provided, however it is possible that increasing oxygen saturation does not completely overcome the underlying metabolic changes associated with hypoxemia, leading to misclassification and type II error. Fourth, our study looked at serum samples taken immediately preoperatively, which may not necessarily reflect the general pattern of metabolite expression over time, but rather represents a discrete time point. We were also limited in this study to categorization by immediate preoperative saturations, therefore both the duration and average levels of hypoxemia in these patients could vary. In addition, this study used secondary samples and was not primarily designed or powered for these measured outcomes. Instead, this secondary study provides key preliminary data to identify potential metabolic changes of interest that can be targeted as the primary outcomes of future observational and mechanistic studies. Importantly, as was previously mentioned, the biologic relevance of our findings remains to be determined. Since metabolomics is a novel approach to the study of CHD, there are no data available in this population with which to compare our findings. Other studies that looked at low oxygen states (not in CHD, but *in vitro* or in other patient populations with hypoxemia) have demonstrated similar findings and researchers have proposed mechanisms that could explain the metabolite alterations seen in a hypoxemic state. These studies, as well as our own findings, can help inform future studies that focus solely on the metabolites that we found to be of potential biologic relevance. Next steps would include complete quantitative mapping of these candidate pathways followed potentially by pathway manipulation in *in vitro* or animal models, a staged approach to metabolomics research described by Johnson et al. [14]. Another limitation of our study includes the lack of data available in the long-term post-operative period. Changes in the metabolic state in the immediate post-operative period are difficult to interpret, since CPB is such a substantial physiologic stressor with overwhelming changes to the metabolome that obscure differences found at baseline [4]. However, repeating these serum analyses in patients once their metabolic state has returned to equilibrium would be helpful and may allow us to determine if the anatomic and/or physiologic changes made during surgery actually lead to a true change in a patient’s metabolism.

## 5 Conclusions

In this hypothesis-generating study we found several metabolites that appear to be altered in hypoxemic neonates with CHD. This study has generated 12 potential metabolic targets that could be explored in mechanistic studies, specifically quantifying serum metabolite levels in hypoxemic neonates, and the levels of both upstream and downstream metabolites in their respective pathways, in order to determine changes in enzymatic activity as a result of hypoxemia. More detailed pathway analysis, focused on the aforementioned pathways that appear to be altered in our hypoxemic neonates, would be useful in order to better understand how hypoxemia leads to the changes we see in our neonatal cohort.

## Figures and Tables

**Table 1:** Baseline characteristics, pre-operative oxygen saturation, respiratory support, hematocrit, and diagnosis of the entire cohort, and of neonates and infants separately

	Entire Cohort (n = 82)	Neonates (n = 42)	Infants (n = 40)	*p*-value
**Female; n (% of cohort)**	37 (45.1%)	22 (52.4%)	15 (37.5%)	0.19
**Age-days; median (range)**	20 (1, 120)	5.5 (1, 22)	82 (32, 120)	<0.0001
**Weight in kg; median (range)**	3.6 (2.1, 7.9)	3.1 (2.1, 4.0)	4.5 (2.7, 7.9)	<0.0001
**Preterm; n (% of cohort)**	13 (15.9%)	3 (7.1%)	10 (25%)	0.04
**SpO**_**2**_ **≤92%; n (% of cohort)**	42 (51.2%)	24 (57%)	18 (45%)	0.38
**Type of respiratory support; n (% of cohort)**	None–47 (57.3%)	24 (57.1%)	23 (57.5%)	0.29
	NC–23 (28%)	9 (21.4%)	14 (35%)	
	NIPPV–4 (4.9%)	3 (7.1%)	1 (2.5%)	
	Intubated–8 (9.8%)	6 (14.3%)	2 (5%)	
**Hematocrit; median (range)**	38.7 (26, 55.7)	40.8 (28.4, 55.7)	35.6 (26, 45.1)	<0.0001
**Diagnosis (n, % of cohort)**		HLHS (17, 40%)	TOF (9, 23%)	N/A
		VSD/Arch (7, 17%)	VSD (8, 20%)	
		TGA (5, 12%)	DORV (4, 10%)	
		Truncus Arteriosus (4, 10%)	AVSD (3, 8%)	
			PA/VSD (2, 5%)	
		PA/VSD (3, 7%)	VSD/Arch (2, 5%)	
		DORV (2, 5%)	HLHS (1, 3%)	
		Arch (1, 2%)	TAPVR (1, 3%)	
		PA/IVS (1, 2%)	Truncus Arteriosus (1, 3%)	
		Other (2, 5%)		
			Other (9, 23%)	

HLHS indicates hypoplastic left heart syndrome; VSD/Arch, ventricular septal defect with coarctation of the aorta; TGA, transposition of the great arteries; PA/VSD, pulmonary atresia with ventricular septal defect; DORV, double outlet right ventricle; Arch, coarctation of the aorta; PA/IVS, pulmonary atresia with intact ventricular septum; AVSD, atrio-ventricular septal defect; TAPVR, total anomalous pulmonary venous return; TOF, Tetralogy of Fallot.

## Abbreviations

5-MTP: 5-methoxytryptophan
AMP: adenosine monophosphate
CHD: congenital heart disease
CNS: central nervous system
CPB: cardiopulmonary bypass
Epo: erythropoietin
FDR: false discovery rate
LTIH: long-term intermittent hypoxia
MMA: methylmalonic acid
PD-ECGF: platelet-derived endothelial cell growth factor
PLS-DA: partial least squares discriminant analysis
RBC: red blood cell
ROS: reactive oxygen species
SpO_2_: oxygen saturation
TP: thymidine phosphorylase
VIP: variable importance in projection
XO: xanthine oxidase
